# Determination of ITS1 haplotypes of Fritillariae Cirrhosae Bulbus by amplicon sequencing

**DOI:** 10.1186/s13020-024-00911-3

**Published:** 2024-02-28

**Authors:** Hoi-Yan Wu, Ka-Lok Wong, Sean Tsz-Sum Law, Wenyang Nong, Kwun-Tin Chan, Jerome Ho-Lam Hui, Ge Lin, Wing-Han Chan, Pang-Chui Shaw

**Affiliations:** 1 Li Dak Sum Yip Yio Chin R & D Centre for Chinese Medicine, The Chinese University of Hong Kong, Shatin, N.T., Hong Kong China; 2 Government Chinese Medicines Testing Institute, Chinese Medicine Regulatory Office, Department of Health, Shatin, N.T., Hong Kong China; 3 School of Life Sciences, The Chinese University of Hong Kong, Shatin, N.T., Hong Kong China; 4 State Key Laboratory of Research on Bioactivities and Clinical Applications of Medicinal Plants (The Chinese University of Hong Kong) and Institute of Chinese Medicine, The Chinese University of Hong Kong, Shatin, N.T., Hong Kong China; 5 School of Biomedical Sciences, The Chinese University of Hong Kong, Shatin, N.T., Hong Kong China

**Keywords:** Fritillariae Cirrhosae Bulbus, *Fritillaria cirrhosa*, Liliaceae, PCR–RFLP, Haplotypes, Internal transcribed spacer, Amplicon sequencing

## Abstract

**Background:**

Fritillariae Cirrhosae Bulbus is an antitussive and expectorant Chinese medicinal material derived from the dried bulbs of six *Fritillaria* species. In the 2015 edition of the Chinese Pharmacopoeia, the polymerase chain reaction-restriction fragment length polymorphism (PCR–RFLP) is the officially listed method for their authenfication. Specifically, the ~ 300-bp ITS1 amplicon of only Fritillariae Cirrhosae Bulbus but not other *Fritillaria* species can be cleaved into two smaller fragments with restriction enzyme SmaI. Considering repeated reported cases of incomplete digestion of ITS1 amplicon, this study aims to investigate the possibility of heterogeneous ITS1 sequences contained in the Fritillariae Cirrhosae Bulbus.

**Methods:**

In this study, ITS1 amplicons of Fritillaria Cirrhosae Bulbus and four other *Fritillaria* species were sequenced on Illumina platform. We utilised high-throughout amplicon sequencing to determine ITS1 haplotypes and their frequencies in *Fritillaria* genomes.

**Results:**

Our results showed that all six botanical sources of Fritillariae Cirrhosae Bulbus indeed possess ITS1 haplotypes with no SmaI restriction site, and the average percentages of ITS1 reads containing SmaI restriction site ranged from 63.60% to 91.81%.

**Conclusion:**

Our findings suggest that the incomplete digestion in PCR–RFLP analysis of Fritillariae Cirrhosae Bulbus is caused by the presence of ITS1 haplotypes without SmaI restriction site due to intragenomic heterogeneity.

**Supplementary Information:**

The online version contains supplementary material available at 10.1186/s13020-024-00911-3.

## Background

Fritillariae Bulbus (Beimu) has long been used as an antitussive and expectorant herb. Its ethnopharmacological use was first documented in Shennong Bencao Jing [1], the earliest classic text of Chinese materia medica in China compiled in the Eastern Han Dynasty (25–220 AD) [2]. Among various types of Fritillariae Bulbus, Fritillariae Cirrhosae Bulbus (FCB) is more valuable and regarded as “top grade” [3]. FCB has been officially documented as the bulbs of six *Fritillaria* species (*F. cirrhosa* D.Don, *F. unibracteata* P.K.Hsiao & K.C.Hsia, *F. przewalskii* Maxim. ex Batalin, *F. delavayi* Franch., *F. taipaiensis* P.Y.Li and *F. unibracteata* var*. wabuensis* (S.Y.Tang & S.C.Yueh) Z.D.Liu, Shu Wang & S.C.Chen) in the Pharmacopoeia of the People’s Republic of China (Chinese Pharmacopoeia) (2020 edition). DNA technique is an independent approach to traditional species identification methods such as morphological and chemical analysis. DNA test results are not affected by ages, physiological conditions and habitats of organisms, which is particularly useful for discrimination of morphologically confused CMMs and CMMs without unique chemical markers. Compared with DNA sequencing-based methods like DNA barcoding, the experimental procedure of polymerase chain reaction-restriction fragment length polymorphism (PCR–RFLP) is relatively simple and suitable for rapid screening of medicinal materials. PCR–RFLP method for identification of FCB was first included in the First Supplement of the Chinese Pharmacopoeia (2010 edition). It is the first plant-derived materia medica with a DNA-based identification method in the Chinese Pharmacopoeia and also in Hong Kong Chinese Materia Medica Standards (HKCMMS).

The PCR–RFLP method involves the amplification of a ~ 300 bp-fragment from the internal transcribed spacer 1 (ITS1) in the nuclear ribosomal DNA region, followed by the restriction digestion by SmaI restriction enzyme. As SmaI restriction site (5′-CCCGGG-3′) is present in the ITS1 region of FCB but not in that of other Fritillariae Bulbus (non-FCB), only FCB species would give one ~ 200-bp and one ~ 100-bp fragments after SmaI digestion. For non-FCB, there should be only one ~ 300-bp fragment after digestion, or no band at all because of the absence of PCR amplicon. These unique RFLP patterns allow the differentiation of FCB from the bulbs of other *Fritillaria* species qualitatively [4]. However, it has long been known that the 300-bp ITS1 amplicon of FCB may not be completely cut, and weak, uncut 300-bp bands could be observed after PCR–RFLP in various studies [4–7]. This incomplete digestion might limit the applicability of the PCR–RFLP method towards processed FCB samples, such as FCB powder. The uncut 300-bp could be ambiguous as the operator is unable to determine whether its presence is due to admixture of non-FCB species or is just a natural phenomenon in some FCB species.

The internal transcribed spacers (ITSs) lie within the 35S ribosomal DNA units. ITS1 and ITS2 are transcribed but non-coding sequences between the 18S and 5.8S rRNA genes (ITS1) and between the 5.8S and 25S/28S rRNA genes (ITS2) in eukaryotes [8, 9]. Ribosomal DNA (rDNA) is abundant in eukaryotic genomes with highly variable copy number per genome in different species. The rDNA cistrons exist as arrays of tandem repeats. In plants, the number of rDNA copy varies from 500 to 40,000 per diploid cell [10]. There is a strong positive correlation between rDNA copy number and genome size in both plants and animals [11]. Genus *Fritillaria* is known to carry giant genomes, with genome size values ranging from 30.15 Gb to 85.38 Gb in different species [12]. The copy number of 35S rDNA of *Fritillaria imperialis* was estimated to be around 4000 by Southern blot hybridization or around 6200 by high throughput sequencing [13]. It was once presumed that the sequences of the rDNA copies in the same cell should remain largely the same caused by a sequence homogenization mechanism under concerted evolution [14, 15]. Nonetheless, intragenomic heterogeneity of rDNA sequences (and ITS sequences) exists. With the development of high throughput sequencing, intragenomic variations in rDNA cistrons and ITS sequences have been reported in various groups of fungi [16–18], animals [19–21] and plants [22–24]. Intragenomic heterogeneity of ITS1 sequences has also been reported in *Lilium* and *Tulipa* [22, 25], which belong to Liliaceae, the same family of *Fritillaria.*

In view of the above reasons, it is speculated that *Fritillaria* would also carry a relatively high copy number of rDNA with intragenomic sequence variations, which may cause the incomplete digestion of the 300-bp amplicon in the PCR–RFLP method. We decided to carry out high-throughput amplicon sequencing on *Fritillaria* using Illumina platform to look into the following questions: (1) Is the incomplete digestion on FCB due to the intragenomic heterogeneity of ITS1 sequences, or non-targeted amplification of environmental sequences, such as fungal ITS1? (2) Do different FCB species have different proportion of ITS1 sequences without SmaI restriction site 5′-CCCGGG-3′? (3) How many ITS1 haplotypes do the *Fritillaria* species have? (4) Would FCB and non-FCB species share the same ITS1 haplotypes?

## Methods

### Sample collection

A total of 43 dried bulb samples from 12 *Fritillaria* species were collected from different parts of China or obtained from the curations of Hong Kong Chinese Materia Medica Standards (HKCMMS) program, including 7 samples of *F. cirrhosa* (川貝母), 5 samples of *F. unibracteata* (暗紫貝母), 5 samples of *F. przewalskii* (甘肅貝母), 5 samples of *F. delavayi* (梭砂貝母), 4 samples of *F. taipaiensis* (太白貝母), 2 samples of *F. unibracteata* var. *wabuensis* (瓦布貝母), 3 samples of *F. ussuriensis* (平貝母), 2 samples of *F. pallidiflora* (伊犁貝母), 1 sample of *F. thunbergii* (浙貝母), 4 samples of *F. hupehensis* (湖北貝母), 1 sample of *F. walujewii* (新疆貝母), and 1 sample of *F. puqiensis* (蒲圻貝母) (Table 1). One sample of *F. unibracteata*, RD176, acted as an extraction positive control for DNA extraction and PCR–RFLP procedure, and was included in each batch of experiments. It was also regarded as a sample for amplicon sequencing. Reference material of *F. unibracteata* (T5177) from National Institutes for Food and Drug Control (NIFDC), China, was included for comparison. The collected samples have been authenticated by Prof. Shu Wang of Sichuan University or experts in research team of Prof. Karl Wah-Keung Tsim of Hong Kong University of Science and Technology. Voucher specimens of the samples were deposited at Li Dak Sum Yip Yio Chin R & D Centre for Chinese Medicine, The Chinese University of Hong Kong.

**Table 1:** Information of Fritillariae Bulbus samples

Scientific name	Sample code	Sample mark	Sequencing sample	Source/collection location^a^
Fritillariae Cirrhosae Bulbus (FCB)				
*F. cirrhosa*	T4971	T4971-A		Mao, Sichuan
(川貝母)		T4971-B	Y	
	T4972	T4972-A	Y	Mao, Sichuan
		T4972-B		
	T4973	T4973-A		Mao, Sichuan
		T4973-B	Y	
	RD185	RD185-A		Kangding, Sichuan
		RD185-B	Y	
	RD194	RD194-A		Hong Kong SAR
		RD194-B	Y	
	RD188	RD188-A	Y	Datong, Qinghai
		RD188-B		
	T5223	T5223-A	Y	Somang, Sichuan
		T5223-B		
*F. unibracteata*	T4974	T4974-A	Y	Songpan, Sichuan
(暗紫貝母)		T4974-B		
	T4992	T4992-A		Qinghai
		T4992-B	Y	
	RD181	RD181-A		Hong Kong SAR
		RD181-B	Y	
	RD177	RD177-A		Songpan, Sichuan
		RD177-B	Y	
	T5224	T5224-A		Hongyuan, Sichuan
		T5224-B	Y	
*F. przewalskii*	T4979	T4979-A		Huangzhong, Qinghai
(甘肅貝母)		T4979-B	Y	
	T5230	T5230-A		Gannan, Gansu
		T5230-B	Y	
	T5231	T5231-A		Zhang County, Gansu
		T5231-B	Y	
	T5232	T5232-A		Min, Gansu
		T5232-B	Y	
	T5233	T5233-A	Y	Zhuoni, Gansu
		T5233-B		
*F. taipaiensis*	T4993	T4993-A	Y	Own collection, location unknown
(太白貝母)		T4993-B		
	T5227	T5227-A		Chongqing
		T5227-B	Y	
	T5228	T5228-A	Y	Chongqing
		T5228-B		
	T5229	T5229-A	Y	Chongqing
		T5229-B		
*F. delavayi*	T4977	T4977-A	Y	Qamdo, Tibet
(梭砂貝母)		T4977-B		
	T4978	T4978-A		Yushu, Qinghai
		T4978-B	Y	
	T5234	T5234-A	Y	Ganzi, Sichuan
		T5234-B		
	T5235	T5235-A	Y	Yushu, Qinghai
		T5235-B		
	T5236	T5236-A		Gongga, Tibet
		T5236-B	Y	
	T5237	T5237-A		Naqu, Tibet
		T5237-B	Y	
*F unibracteata*	T4975	T4975-A	Y	Mao, Sichuan
var. *wabuensis*		T4975-B		
(瓦布貝母)	T4976	T4976-A		Mao, Sichuan
		T4976-B	Y	
Congeneric *Fritillaria* species (non-FCB)				
*F. ussuriensis*	T4980	T4980-A	Y	Shangzhi, Heilongjiang
(平貝母)		T4980-B		
	T5221	T5221-A		Hong Kong SAR market
		T5221-B	Y	
	T5225	T5225-A		Jilin
		T5225-B	Y	
*F. thunbergii*	T4981	T4981-A	Y	Zhejiang
(浙貝母)		T4981-B		
*F. hupehensis*	T4982	T4982-A	Y	Enshi, Hubei
(湖北貝母)		T4982-B		
	RD169	RD169-A		Enshi, Hubei
		RD169-B	Y	
	RD170	RD170-A	Y	Enshi, Hubei
		RD170-B		
	T4940	T4940-A		Own collection, location unknown
		T4940-B	Y	
*F. pallidiflora*	T5239	T5239-A		Own collection, location unknown
(伊犁貝母)		T5239-B	Y	
*F. walujewii*	T4985	T4985-A		Xinjiang
(新疆貝母)		T4985-B	Y	
*F. pallidiflora*	RD216	RD216-A		Yili, Xinjiang
(伊犁貝母)		RD216-B	Y	
*F. puqiensis*	T5240	T5240-A		Own collection, location unknown
(蒲圻貝母)		T5240-B	Y	
*F. cirrhosa*	RD176	RD176-A		Hongyuan, Sichuan
(川貝母)		RD176-B	Y	(A voucher specimen of
*As positive control*		RD176-C		*F. cirrhosa*
		RD176-D		in Hong Kong Chinese
		RD176-E		Materia Medica Standards)
		RD176-F		
		RD176-G		
		RD176-H		
*F. unibracteata*	T5177	T5177-A		Reference material from
(暗紫貝母)		T5177-B		National Institutes for
*As positive control*		T5177-C	Y	Food and Drug Control, China
		T5177-D		(121,000–201609)
		T5177-E		
		T5177-F		

^a^It shows where the samples were bought or collected, but did not necessarily reflect the places of origin of the samples

### DNA extraction

Genomic DNA was extracted using Broad-spectrum Plant Rapid Genomic DNA kit (Biomed, Beijing, China) with modifications. In brief, 50 mg of dried bulb sample was weighed and ground into powder using Mixer Mill MM 400 (Retsch, Nordrhein-Westfalen, Germany) at a shaking frequency of 28 Hz for 30 min. Powdered sample was mixed with 600 μl Lysis Buffer AP1 and 6 μl RNase A before being incubated at 65 °C for 1 h with intermittent vortexing. Then, 190 μl Buffer AP2 was added, and the sample was incubated at – 20 °C for 30 min, followed by 10-min centrifugation at 13,000 rpm. Clear supernatant was obtained and mixed with 900 μl Binding Buffer AP3, before being added to a spin column to purify the DNA, which was eluted in 50 μl Elution Buffer, after two rounds of membrane washing with 500 μl Wash Buffer. The quantity of genomic DNA was measured using a NanoDrop Lite Spectrophotometer (Thermo Fisher Scientific, CA, USA). All samples were extracted in duplicates.

### PCR–RFLP

The polymerase chain reaction-restriction fragment length polymorphism (PCR–RFLP) method for identification of Fritillariae Cirrhosae Bulbus in the Chinese Pharmacopoeia (2020 edition) was modified and performed in duplicates. A 30 μl PCR reaction mixture contained 6 μl 5X PCR buffer (with 2 mM MgCl_2_ at final concentration), 0.6 μl 10 mM dNTPs, 0.5 μl 30 μM forward primer (5′-CGTAACAAGGTTTCCGTAGGTGAA-3′), 0.5 μl 30 μM reverse primer (5′-GCTACGTTCTTCATCGAT-3′), 0.2 μl Q5 High-Fidelity DNA polymerase and 1 μl DNA template. PCR amplification was carried out in a T100 thermal cycler (Bio-Rad, CA, USA) programmed with a pre-denaturation at 95 °C for 4 min, 30 cycles of 30 s at 95 °C, 30 s at 55 °C and 30 s at 72 °C, followed by a final extension at 72 °C for 5 min, as stated in the Chinese Pharmacopoeia. The PCR products were subjected to RFLP according to Chinese Pharmacopoeia. A 20 μl reaction containing 1X CutSmart buffer, 6 μl PCR product and 5U SmaI (NEB, MA, USA) was incubated at 30 °C for 2 h. Results were visualized by 1.5% agarose gel electrophoresis.

### Illumina MiSeq amplicon sequencing

Sequencing libraries were generated with reference to “16S Metagenomic Sequencing Library Preparation” for Illumina MiSeq Platform [26]. The first-stage PCR involves amplification of the ITS1 region using a pair of primers with adaptors added to the 5′ ends of the primers for Fritillariae Cirrhosae Bulbus in Chinese Pharmacopoeia (BeiMu_Miseq_1F: 5′-TCGTCGGCAGCGTCAGATGTGTATAAGAGACAGGCTACGTTCTTCATCGAT -3′ and BeiMu_Miseq_1R: 5′-GTCTCGTGGGCTCGGAGATGTGTATAAGAGACAGCGTAACAAGGTTTCCGTAGGTGAA-3′) using Q5 High-Fidelity DNA polymerase. The ITS1 amplicons were indexed in the second-stage PCR using Nextera XT index kit v2 Set A and purified according to the manufacturer’s instructions. The prepared libraries were quantified using Qubit 2.0 Fluorometer. Normalized libraries were pooled and sequenced with an Illumina MiSeq platform using the 2 × 300 bp paired end protocol.

### Amplicon sequence variants analysis

Raw sequencing reads were first demultiplexed and then analysed using the DADA2 package in R [27]. Primers were removed from the reads with *Cutadapt*. Trimming and filtering was performed using *filterAndTrim* function with maxN at 0, maxEE at c(5, 5), truncQ at 2, maxEE of 5 and minLen at 80. The forward and reverse reads were denoised and merged into paired reads. Chimera sequences were then removed with de novo chimera checking with default *consensus* option. The resulting amplicon sequence variants (ASVs, i.e. haplotypes) were then outputted in a ASV table summarising the sequence of each ASV and the abundance of the ASVs in different samples. To separate the ASVs of ITS1 of *Fritillaria* species from those of fungi, the ASVs were mapped to the ITS1 sequence, obtained by Sanger sequencing, of the reference material of *F. unibracteata* (T5177) from NIFDC using *clc_mapper* function of CLC Assembly Cell package v5.1.1. Mapped ASVs with length longer than 250 bp were regarded as full-length ASVs of ITS1 region of *Fritillaria* species. Only full-length ASVs with over 1% abundance in at least one of the samples were regarded as major ASVs and used for further analysis. To detect the fungal ITS1 amplicons, the *assignTaxonomy* function, a naïve Bayes classifier (RDP classifier) implemented in DADA2, was carried out using UNITE general FASTA release for Fungi “sh_general_release_dynamic_10.05.2021.fasta” database [28].

### p-distance analysis

All major ASVs were aligned using MAFFT [29]. MEGA X [30] was used to calculate p-distances between all major ASVs for intragenomic, intraspecific and interspecific comparisons.

### Haplotype network analysis

For validation, the major ASVs were aligned with the ITS1 sequences from the *F. unibracteata* reference (T5177) and 7 *Lilium* species as outgroup, which were retrieved from Rønsted et al. (2005) [31], using MAFFT [29]. The aligned sequences were used to construct a maximum likelihood tree using FastTree [32], which was then visualized in Evolview v3 [33]. Subsequently, a haplotype network for major ASVs was constructed under TCS method [34] using PopART version 1.7 [35] with Ɛ value set at 0. To reduce the computer processing demand while maintaining the relative abundance of the major ASVs, we divided the abundance of the major ASVs by 100 in all samples. Graphic editing was performed with Inkscape Vector software version 1.1.2 (https://www.inkscape.org/).

## Results

### PCR–RFLP patterns

**Fig. 1:**
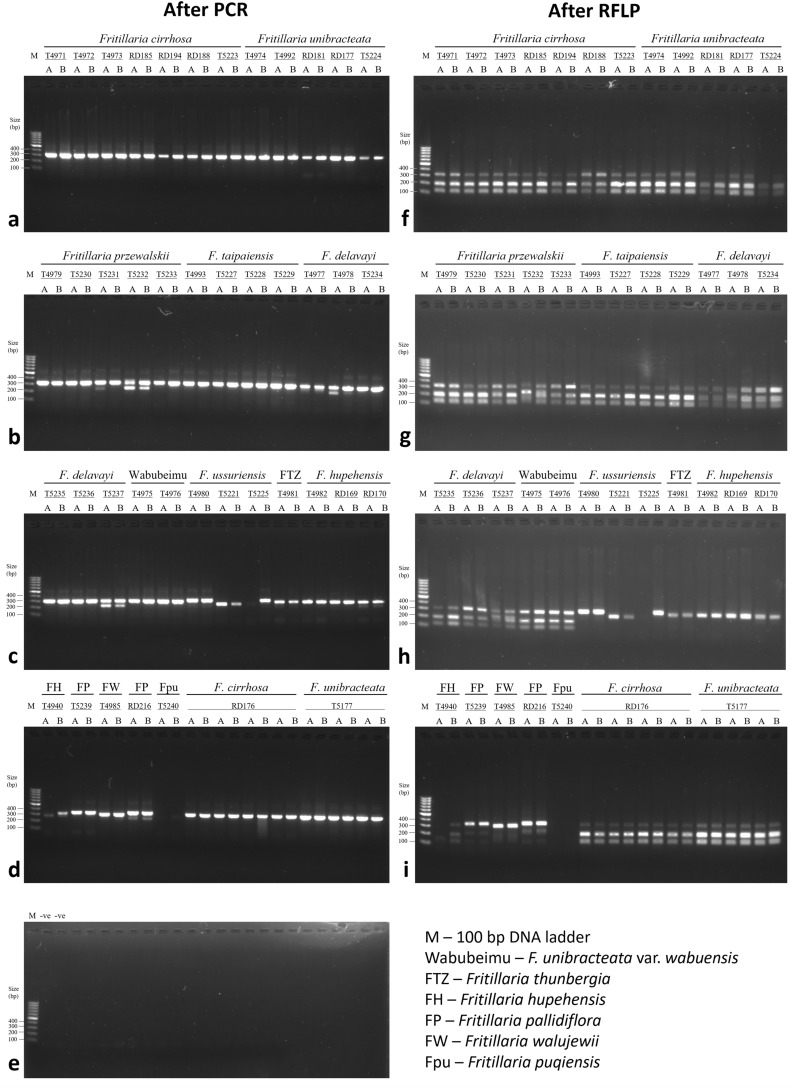
PCR–RFLP of genuine and non-genuine *Fritillaria* species. All six genuine species could be successfully amplified and digested, with weak 300-bp bands remaining uncut. The non-genuine samples were either not amplified or produced a ~ 300-bp band that was only weakly digested. RD176 and T5177 were chosen as extraction positive controls and were extracted together with the samples in different rounds of extractions

All samples were analyzed in duplicates for PCR–RFLP assay. One sample of *F. cirrhosa*, RD176, was included in each batch of extraction as an extraction positive control. Results of PCR–RFLP assay before and after SmaI digestion are shown in Fig. 1a–e and f–i, respectively. We loaded 10 μl RFLP products per well, relatively high amounts compared to recommended levels stated in the monograph of Fritillariae Cirrhosae Bulbus of the Chinese Pharmacopeia, for better visualization of non-specific amplification and undigested bands. Clear and bright 300-bp bands of varying intensity were obtained from all FCB samples after PCR (Fig. 1a–c). In sample T5231-A (*F. przewalskii*), T5232-A and -B (*F. przewalskii*), T4978-A and -B (*F. delavayi*), T5237-A and -B (*F. delavayi*), non-targeted amplicons at about 250 bp were observed. Most of the non-FCB also gave a 300-bp band, except for T5225-A (*F. ussuriensis*), T4940-A (*F. hupehensis*) and T5240-A and -B (*F. puqiensis*), which gave no band or very weak bands of different sizes.

After SmaI digestion, ~ 200-bp band and ~ 100-bp band were seen in all FCB samples, which conform to the positive results of authentic Fritillariae Cirrhosae Bulbus. However, the 300-bp band of different degree of brightness remained in all FCB samples. For congeneric non-FCB, the amplicons of *F. ussuriensis* and *F. thunbergii* were not digested. Digested bands of ~ 200 bp and ~ 100 bp were observed in T4940-B (the sample claimed to be *F. hupehensis)*, T5239-A and -B and RD216-A and -B (*F. pallidiflora*) (Fig. 1i), with the digested bands of T5239-A and -B being weaker than the others.

### Determination of ASVs

For each sample, the DNA extract duplicate that gave a stronger 300-bp band after PCR was selected for amplicon sequencing. The data of high throughput pair-end amplicon sequencing and the results of amplicon sequence variants (ASVs) picking are summarized in Table 2. In total, 13,386–220,794 (82,719 on average) raw reads were generated per sample. After filtering, denoising, pair-read merging and removal of chimera by DADA2, there were 6353–76,376 (31,950 on average) denoised, merged, non-chimera reads per sample. All ASVs available and their abundance in different samples were outputted for further analysis. In total, there were 1,373,848 reads, consisting of 2708 ASVs. The resulting ASVs were mapped to the ITS1 sequence of the reference material of *F. unibracteata* (T5177). ASVs successfully mapped to this reference sequence and with a length longer than 250 bp were regarded as full-length *Fritillaria* ITS1 ASVs, or ITS1 haplotypes, with a total number of 1521. T5221-B, T5240-B and T4985-B did not contain any reads that could be mapped to the reference sequence, and therefore were not further analysed. For FCB species, most of their non-chimera reads (82.03% in average) were full-length *Fritillaria* ITS1 sequences, with the exception of T5237-B, T5232-B and T4976-B, which contained only 18.05%, 20.01% and 46.98% full-length *Fritillaria* ITS1 reads, respectively. T5237-B and T5232-B, as mentioned above, had produced a non-specific 250-bp band after PCR, whereas T4976-B had produced a 300-bp band which was only partially digested by SmaI (Fig. 1h). For non-FCB samples, the average percentage of full-length *Fritillaria* ITS1 reads was only 64.28%, indicating a higher level of non-targeted amplification than FCB.

**Table 2:** Statistics of data from high throughput amplicon sequencing

Species	Sample	Raw reads	Filtered	Denoised, merged reads	Non-chimera reads	Mapped to ITS1 of T5177 (*F. unibracteata*)								Identified as fungal ITS		
						# Reads full-length *Fritillaria* ITS1	% Full-length *Fritillaria* ITS1	# Reads belonging to major ASVs	# Total ASVs	# Major ASVs (> 1% frequency)	% Reads w/CCCGGG	% Reads w/CTCGGG	% Reads w/CACGGG	# Fungal reads	% Fungal reads	# Fungal reads > 250 bp
*F. cirrhosa*	RD176-B	83,391	44,487	32,186	28,399	26,921	94.80	26,402	22	8	99.06	0.00	0.00	483	1.70	0
	RD185-B	60,733	37,116	21,945	18,085	17,445	96.46	17,169	16	10	99.35	0.24	0.00	40	0.18	36
	RD188-A	68,403	32,109	20,739	20,229	16,093	79.55	15,991	12	7	75.93	23.73	0.00	3622	17.91	49
	RD194-B	78,909	49,180	33,971	31,511	30,372	96.39	29,960	19	8	99.33	0.09	0.00	1002	3.18	0
	T4971-B	88,324	55,193	31,900	28,616	26,400	92.26	24,232	40	9	79.35	1.49	17.21	103	0.36	0
	T4972-A	80,715	48,411	25,627	24,376	22,595	92.69	20,434	53	8	90.46	1.43	2.70	249	1.02	0
	T4973-B	85,026	43,600	20,698	20,698	19,599	94.69	17,444	34	6	92.12	2.63	0.00	219	1.06	0
	T5223-A	72,061	38,426	23,249	21,082	18,497	87.74	18,206	20	7	98.91	0.39	0.00	739	3.51	12
*F. delavayi*	T4977-A	66,190	39,336	27,737	27,220	16,537	60.75	14,567	47	11	70.96	25.73	0.00	9567	35.15	1107
	T4978-B	220,794	133,813	94,086	76,376	61,465	80.48	56,148	91	9	78.59	19.84	0.00	8993	11.78	0
	T5234-A	69,201	41,430	26,275	23,259	20,727	89.11	19,865	27	6	55.50	42.72	0.00	1264	5.43	78
	T5235-A	78,620	44,957	32,620	26,391	22,837	86.53	22,219	22	4	98.86	0.36	0.00	124	0.47	0
	T5236-B	82,272	50,490	42,057	32,729	32,024	97.85	31,485	23	6	37.11	62.23	0.00	236	0.72	0
	T5237-B	81,155	56,949	49,840	49,804	8990	18.05	8990	4	4	91.71	8.29	0.00	27,984	56.19	12
*F. przewalskii*	T4979-B	94,327	62,946	39,849	32,288	30,621	94.84	29,096	38	10	83.29	14.78	0.35	111	0.34	0
	T5230-B	79,496	45,525	30,384	29,139	28,201	96.78	28,073	13	7	99.70	0.20	0.00	254	0.87	81
	T5231-B	78,000	47,880	28,276	25,094	23,051	91.86	22,686	21	11	91.59	7.48	0.00	1472	5.87	655
	T5232-B	96,145	74,152	63,946	62,846	12,574	20.01	12,461	12	8	81.54	18.36	0.00	49,779	79.21	34
	T5233-A	66,180	39,856	24,691	22,264	19,883	89.31	19,758	17	12	61.22	38.62	0.00	1893	8.50	498
*F. taipaiensis*	T4993-A	107,191	75,478	52,073	50,501	36,432	72.14	33,912	48	4	97.43	1.36	0.00	5761	11.41	0
	T5227-B	79,785	53,985	42,410	34,282	33,642	98.13	33,260	18	4	99.71	0.05	0.00	82	0.24	0
	T5228-A	123,092	76,756	55,043	48,937	38,073	77.80	36,783	30	5	98.28	0.38	0.00	633	1.30	0
	T5229-A	79,453	51,417	33,207	31,562	30,812	97.62	28,845	51	5	96.37	0.95	0.00	116	0.37	0
*F. unibracteata*	RD177-B	65,760	42,786	33,710	28,774	26,390	91.71	25,990	15	5	99.27	0.25	0.00	1047	3.64	41
	RD181-B	77,171	52,119	39,681	31,583	30,819	97.58	29,535	43	6	98.12	0.45	0.06	532	1.68	76
	T4974-A	105,170	60,311	39,083	33,381	32,489	97.33	31,005	27	6	97.34	0.35	0.00	259	0.78	0
	T4992-B	87,918	56,663	39,504	35,196	20,534	58.34	17,129	63	6	91.79	1.49	0.15	5427	15.42	0
	T5177-C	85,432	55,709	37,092	32,032	25,667	80.13	25,174	17	7	99.23	0.00	0.00	4683	14.62	0
	T5224-B	31,220	13,477	10,060	10,060	7983	79.35	6058	42	12	94.45	3.57	0.00	1726	17.16	122
*F. unibracteata* var*. wabuensis*	T4975-A	101,808	69,048	36,232	34,573	32,529	94.09	27,748	82	12	64.50	2.17	27.28	417	1.21	0
	T4976-B	125,753	86,110	46,991	46,603	21,894	46.98	18,303	66	15	62.67	0.00	0.00	21,613	46.38	0
*F. hupehensis*	RD169-B	83,488	35,812	31,038	28,181	26,132	92.73	25,204	42	3	0.00	98.69	0.00	1923	6.82	15
	RD170-A	91,109	44,873	40,561	40,287	23,831	59.15	22,582	25	3	0.48	97.72	0.00	15,707	38.99	178
	T4940-B	50,213	28,361	21,967	21,967	11,400	51.90	8747	57	29	63.81	1.90	27.77	10,126	46.10	236
	T4982-A	57,725	25,230	23,304	20,861	20,309	97.35	18,899	34	3	0.00	98.56	0.00	482	2.31	0
*F. pallidiflora*	RD216-B	112,772	65,531	54,317	52,481	9186	17.50	9109	11	6	8.97	89.81	0.00	43,105	82.13	0
	T5239-B	67,848	19,435	12,719	12,655	8415	66.50	8312	6	1	0.00	99.31	0.00	1556	12.30	91
*F. thunbergii*	T4981-A	71,190	38,222	33,819	30,877	19,170	62.09	16,933	32	4	0.00	96.80	0.00	11,623	37.64	0
*F. ussuriensis*	T4980-A	102,777	44,635	28,471	28,226	10,483	37.14	10,278	11	6	0.00	98.49	0.00	16,622	58.89	0
	T5221-B	57,979	36,340	33,437	33,437	0	X							32,714	97.84	0
	T5225-B	48,768	17,641	14,122	14,122	13,298	94.17	12,993	11	7	0.00	97.44	0.00	168	1.26	0
*F. puqiensis*	T5240-B	13,386	7338	6514	6353	0	X							899	14.15	45
*F. walujewii*	T4985-B	99,985	78,816	74,462	66,511	80	X							66,402	99.84	58,325

There were 1–29 major ASVs in each sample. Major ASVs represented 76.73–100% of full-length *Fritillaria* ITS1 sequences in all samples. Detailed figures of major ASVs were also shown in Table 2. In subsequent analyses, all reads and ASVs of *Fritillaria* ITS1 were used for comparison of sequences in the locus of SmaI recognition site and p-distance analysis, respectively. Major ASVs were included for building phylogenetic tree and haplotype network for better clarity and lower computational demand without sacrificing many details.

### Locus of SmaI recognition sites

The 3 most frequent sequences at the locus of SmaI recognition sites in *Fritillaria* ITS1 reads were CCCGGG, CTCGGG and CACGGG, differing only at the second position. The percentage of *Fritillaria* ITS1 reads containing the recognition site CCCGGG was significantly different between FCB and non-FCB species. For *F. cirrhosa*, *F. taipaiensis*, and *F. unibracteata*, the average percentages were 91.81 ± 9.45%, 97.95 ± 1.41% and 96.70 ± 3.10%, respectively. The percentages of the remaining three FCB species were lower (83.47 ± 14.39% for *F. przewalskii*, 72.12 ± 23.00% for *F. delavayi* and 63.59 ± 1.29% for *F. unibracteata* var. *wabuensis*). Samples of *F. delavayi* varied the most in the percentage of reads containing CCCGGG, from 37.11% (T5236-B) to 98.86% (T5235-A). FCB samples with less than 70% CCCGGG-containing reads also had brighter undigested bands after RFLP (Fig. 1g, h). *Fritillaria* ITS1 reads containing CCCGGG were absent in most non-FCB samples. Sample T4940-B (*F. hupehensis*) and RD216-B (*F. pallidiflora*) were two exceptions. The presence of CCCGGG-containing reads in these two samples were also in line with the presence of digested ~ 200-bp and ~ 100-bp bands in Fig. 1i. T4940-B showed a high percentage of CCCGGG-containing ASVs (63.81%) and it had 29 major ASVs that could not be found in the three other *F. hupehensis* samples. Further, T4940-A was not successfully amplified at all, with an absence of the 300-bp band in Fig. 1d. The results suggested that T4940 might have been misidentified. RD216-B, *F. pallidiflora*, only had 8.97% CCCGGG-containing reads. However, no CCCGGG-containing read could be found in another sample of *F. pallidiflora* (T5239-B). We further looked into the major ASVs of these two samples. All 29 major ASVs of T4940-A were matched to several FCB species in MegaBLAST, suggesting that this sample has been misidentified or mixed into a batch of *F. hupehensis* sample. One out of six major ASVs (Seq151) of RD216-B contained the CCCGGG sequence. However, Seq151 is still matched to *F. pallidifora* (Accession MN121628.1) at 98.94% identity in MegaBLAST. This revealed that non-FCB species could still contain a small proportion of CCCGGG-containing ITS1 haplotypes. The second most common sequence was CTC GGG, which accounted for in average 9.2% and 86.53% of *Fritillaria* ITS1 reads in FCB and non-FCB *Fritillaria* samples, respectively.

### Clustering of ASVs

To estimate the sequence variations among different ASVs within individuals, within species and between different species, we calculated the p-distances between ASVs. Table 3 shows the minimum and maximum values of intragenomic, intraspecific, and interspecific p-distances of each *Fritillaria* species. All six FCB species had the same minimal intragenomic p-distance, 0.0038. Their maximum intragenomic p-distances ranged from 0.0347 to 0.0795 (0.0583 in average). The maximum intragenomic p-distances of non-FCB species were 0.0641–0.2336, significantly higher than those of the FCB species (p = 0.015 in Student’s t-test). The maximum intraspecific p-distances were not significantly higher than those of intragenomic distances (p > 0.05 in Student’s t test), especially for non-FCB species which only had 1–4 individuals. The minimum interspecific p-distances of 8 out of 10 *Fritillaria* species were zero, meaning that those species have shared at least one identical ASV with other species.

**Table 3:** p-distances of ITS1 ASVs in various *Fritillaria* species

	Intragenomic		Intraspecific		Interspecific	
	Min	Max	Min	Max	Min	Max
*F. cirrhosa*	0.0038	0.0712	0.0000	0.1012	0.0000	0.2214
*F. delavayi*	0.0038	0.0471	0.0000	0.0511	0.0000	0.1936
*F. przewalskii*	0.0038	0.0587	0.0000	0.0629	0.0000	0.1975
*F. taipaiensis*	0.0038	0.0347	0.0000	0.0347	0.0000	0.1767
*F. unibracteata* var*. wabuensis*	0.0038	0.0588	0.0000	0.0588	0.0000	0.1913
*F. unibracteata*	0.0038	0.0795	0.0000	0.0795	0.0000	0.1927
*F. hupehensis*	0.0038	0.1394	0.0000	0.1394	0.0000	0.2428
*F. pallidiflora*	0.0179	0.2336	0.0179	0.2336	0.0232	0.2309
*F. thunbergii*	0.0038	0.0641	0.0038	0.0641	0.0000	0.2081
*F. ussuriensis*	0.0035	0.1491	0.0000	0.1491	0.0308	0.2428

**Fig. 2:**
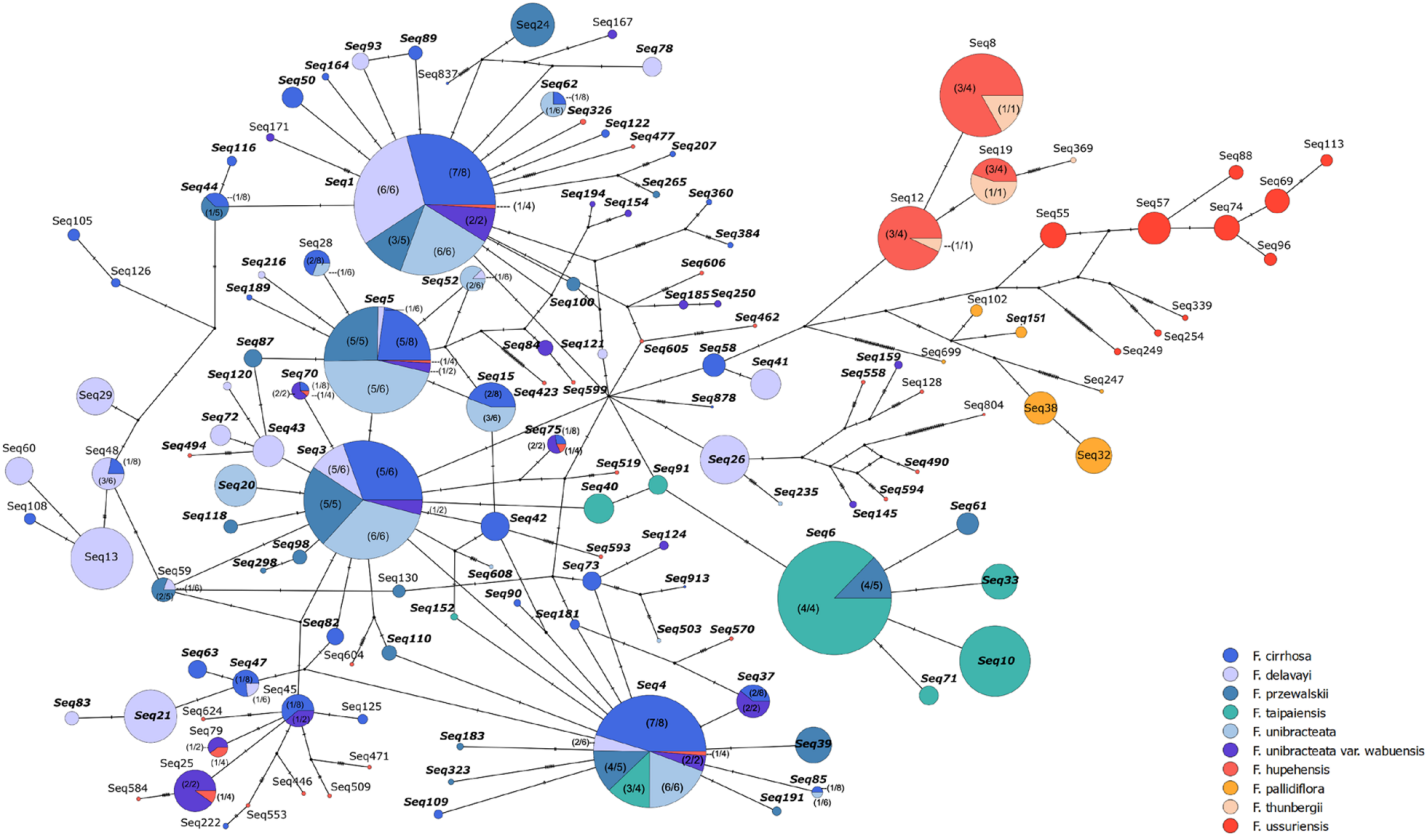
A TCS network constructed for major ASVs (haplotypes) of ITS1 sequences of all *Fritillaria* species investigated. Sizes of the circles are proportional to the number of ASVs. The number of mutations between haplotypes are indicated by the hatch marks. ASVs containing the CCCGGG recognition site are in bold. *F. hupehensis* was found to be grouped into FCB clusters, which was due to the misidentification of T4940 as *F. hupehensis* or T4940 was mixed into a batch of *F. hupehensis* sample

The identities of 139 major ASVs were confirmed by constructing a phylogenetic tree with the ITS1 sequences from the *F. unibracteata* reference (T5177) and 7 *Lilium* species as outgroup (Additional file 1: Fig. S1a). Of all major ASVs, 92 contain the restriction site CCCGGG (Additional file 1: Fig. S1b). Further, the major ASVs of all *Fritillaria* samples were plotted in one haplotype network (Fig. 2), in order to illustrate the relationship among the ASVs and to show how many ASVs were shared among multiple FCB and non-FCB species. The ASVs of FCB and non-FCB species can be generally separated into two clusters. The four most abundant ASVs, namely Seq1, Seq3, Seq4 and Seq5, were present in 5–6 FCB species (Fig. 2; Additional file 1: Fig. S1c, d). It appeared to be more common for FCB species, than non-FCB species, to share the same ASVs. Out of the 95 ASVs in FCB species, 18 of them were present in more than one FCB species. For non-FCB species, only 3 out of 51 ASVs were shared between two species, *F. hupehensis* and *F. thunbergia*. Twenty-eight ASVs belonging to the “FCB” cluster were also present in a non-FCB sample, which was the *F. hupehensis* sample in doubt, T4940-B. Most of them contained the SmaI recognition sequence 5′-CCCGGG-3′. It is also worth noting that the ASVs of FCB species that did not contain the recognition sequence were still in the “FCB” cluster and were linked to the more abundant, CCCGGG-containing ASVs by just several mutational steps. This showed that the alternation of the SmaI recognition sequence was because of the mutations of ASVs of FCB species, rather than the presence of ASVs of non-FCB species.

**Fig. 3:**
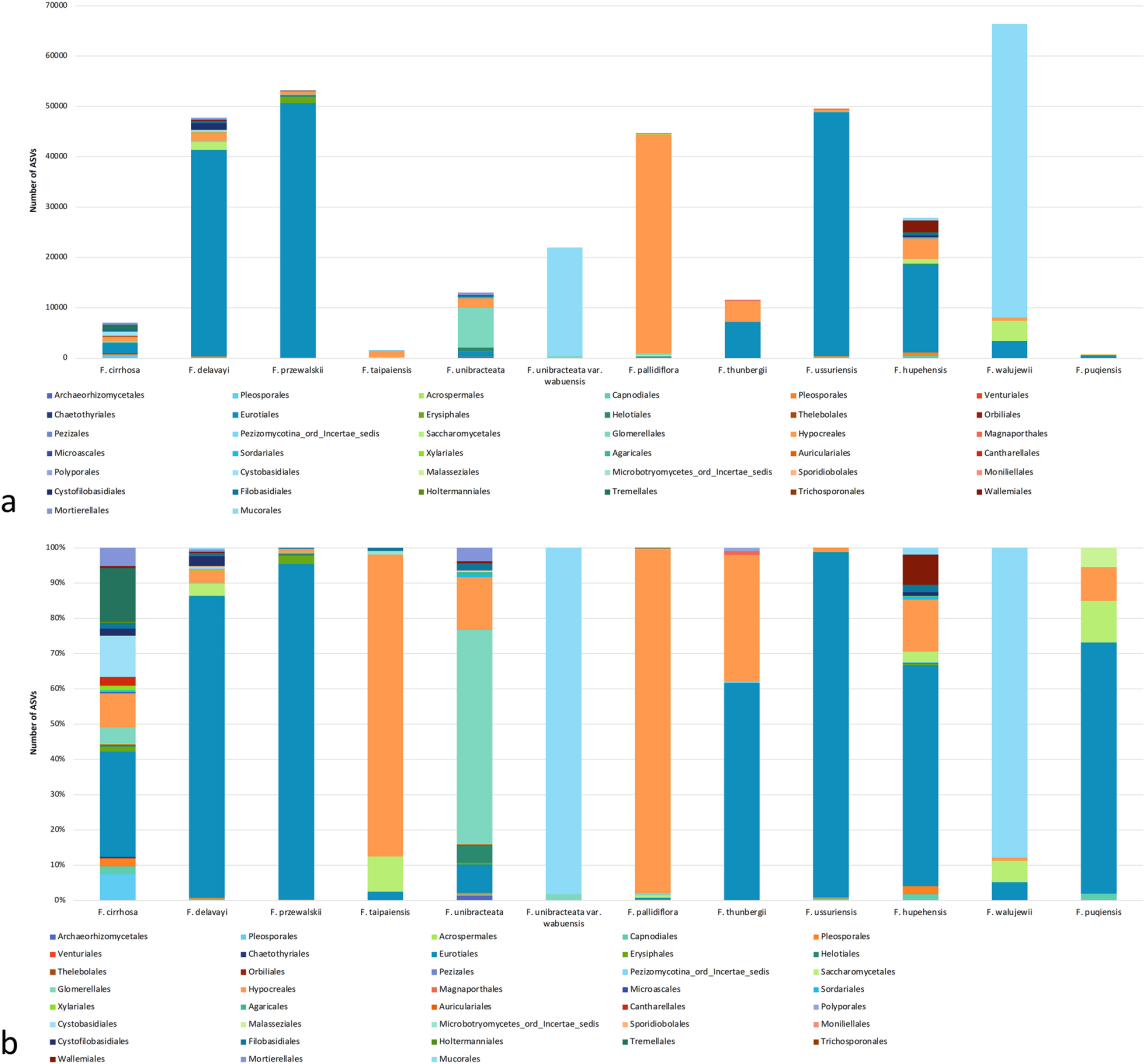
The numbers (**a**) and percentages (**b**) of fungal ASVs identified at order level for each *Fritillaria* species

We attempted to determine if the remaining ASVs that were not mapped as *Fritillaria* ITS1 sequences belonged to fungal ITS, by using the *assignTaxonomy* function implemented in DADA2. As expected, samples with lower percentages of full-length *Fritillaria* ITS ASVs would have higher percentages of ASVs identified as fungal (Table 2). Samples that showed distinct non-target bands at ~ 250 bp in gel electrophoresis after PCR (T5232, T5237, T5221, T4985, RD216) (Fig. 1b–d) showed high percentages of fungal ASVs (over 50%). To investigate whether the ~ 300-bp undigested bands in gel electrophoresis after PCR–RFLP were from the fungal ITS sequences, we counted the number of fungal ASVs with length larger than 250 bp, as the ~ 300-bp amplicons in PCR–RFLP should yield ASVs of approximately 266 bp in length after primer trimming. However, most samples, FCB or non-FCB, did not produce any fungal ASVs longer than 250 bp. For samples that did have fungal ASVs larger than 250 bp, the numbers and proportions relative to the total denoised, merged reads were very small. This showed that the ~ 300-bp undigested bands were not originated from fungal ITS sequences co-amplified with the samples. Some fungal ASVs could be identified up to species level, while some were only identified to genus or family level. The numbers and proportions of fungal ASVs of different orders for each species are shown in Fig. 3.

## Discussion

In this study, we aim to investigate the reason of incomplete digestion of the 300-bp amplicon from FCB in PCR–RFLP assay for the identification of Fritillariae Cirrhosae Bulbus listed in Chinese Pharmacopoeia. We have collected 43 samples from six FCB species and four non-FCB species for PCR–RFLP assay and high-throughput amplicon sequencing of the 300-bp PCR products obtained. The target region of this method has not been stated in the monograph of Fritillariae Cirrhosae Bulbus of the Chinese Pharmacopeia; nevertheless, through our DNA sequence analysis, we revealed that the target region was actually the ITS1 region of *Fritillaria* species. Therefore, we focused on the ITS1 region of *Fritillaria* in this study. We have confirmed the intragenomic heterogeneity of ITS1 sequences, i.e. the presence of multiple ITS1 haplotypes, in various *Fritillaria* species by high-throughput amplicon sequencing. In fact, variations at CCCGGG-recognition site of FCB species could also be confirmed by the minor peaks in the electropherograms produced by Sanger sequencing (Additional file 2: Fig. S2).

ITS1 haplotypes without CCCGGG-recognition site were found in all FCB species, while CCCGGG-containing ITS1 haplotype could be found in one non-FCB sample (*F. pallidifora*, RD216-B). By eliminating any contribution to the 300-bp band from fungal ITS1 amplicons, we showed that the incomplete digestion in FCB species in the PCR–RFLP assay was due to the intragenomic heterogeneity of ITS1 sequence. We believe that this situation is not an isolated incident, as incomplete digestion of PCR amplicon could also be observed in a PCR–RFLP identification assay for *Pulsatilla chinensis* based on ITS2 sequences [36].

The number of full-length *Fritillaria* ITS1 ASVs and the genetic distances among them can indicate the divergence of the region within and among different genomes [22]. In this study, we found that each sample contained 4–91 full length *Fritillaria* ITS1 ASVs, suggesting that all *Fritillaria* samples exhibited intragenomic heterogeneity in ITS1 sequences with different numbers of ITS1 haplotypes. Similar to a previous study [37], only ASV with an emergence frequency above 1% in at least one of the samples would be regarded as major ASV. A high proportion of reads mapped to *Fritillaria* suggesting that the PCR amplification of ITS1 of FCB *Fritillaria* species was quite successful with a low proportion of non-targeted amplicons. It should be noted that the fungal identification was based on ITS sequences amplified with a pair of ITS primers targeting *Fritillaria*, which was not universal to fungus in general. A successful amplification of *Fritillaria* ITS sequences would, expectedly, have very little or no co-amplification of fungal ITS sequences. The fungal species identified in this study were merely those co-amplified in the PCR–RFLP assay. They do not represent the compositions of the fungal microbiome associated with the *Fritillaria* bulbs.

Our results should raise concern on selection of multi-copy DNA regions, such as ITS1 and ITS2, as genetic markers for developing molecular identification method. Apart from differentiation power between species, i.e. specificity, sensitivity and cross reactivity of the assay, intragenomic variation of genetic marker should also be taken into consideration during method development. The haplotype network (Fig. 2) illustrates that several ITS1 ASVs/haplotypes are shared among multiple *Fritillaria* species. Similar ITS2-haplotype sharing has also been previously reported in other plants [22, 37]. Whether this phenomenon would lead to false positive identification or over-estimation of the number of plant species in molecular authentication of multi-herb products remain to be investigated [38]. This study has demonstrated how the intragenomic heterogeneity of a multi-copy genetic marker would lead to ambiguous results in an identification test and limit the applicability of the test to qualitative identification of sample originated from one species only.

## Conclusion

In summary, our research confirms that FCB possesses ITS1 haplotypes with no SmaI restriction site. Moreover, different FCB species have different proportion of ITS1 sequences without the restriction site. Overall, this study contributes to the investigation of a scientific approach in explaining incomplete digestion in PCR–RFLP analysis and strategy could aid in the development of DNA test for identification of Chinese herbal medicine.

### Supplementary Information

**Additional file 1: Fig. S1.** a) Maximum likelihood tree of ITS1 sequences from *Fritillaria* major ASVs and the T5177 reference sequence (highlighted in green) as well as 7 *Lilium* species (highlighted in orange); b) Loci of SmaI recognition site CCCGGG and its mutated forms of CTCGGG, CCACGGG and others, indicated by green, yellow, blue and grey, respectively; c) heatmap showing presence or absence of major ASVs in Fritillariae Cirrhosae Bulbus (FCB) and non-FCB species. *F. hupehensis* was found to be carried major ASVs with SmaI recognition site CCCGGG, which was due to the misidentification of T4940 as *F. hupehensis* or T4940 was mixed into a batch of *F. hupehensis* sample; d) heatmap showing the relative abundance of major ASVs in each FCB and non-FCB species.

**Additional file 2: Fig. S2.** Sanger sequencing electropherograms of the SmaI restriction site in the ITS1 region of Fritillaria Cirrhosae Bulbus (FCB) species. Minor variants in SmaI restriction sites (CCCGGG) of selected FCB samples, including RD188 of *F. cirrhosa* (a), T4975 of *F. unibracteata* var. *wabuensis* (b), T5233 of *F. przewalskii* (c), T5234 of *F. delavayi* (d) and T5236 of *F. delavayi* (e), were observed in the corresponding electropherograms. The minor peaks shown are in line with the high throughput sequencing results reported in Table 2.

## Data Availability

The datasets used or analyzed throughout this study are available from the corresponding author upon reasonable request.

## Abbreviations

FCB: Fritillariae Cirrhosae Bulbus
PCR–RFLP: Polymerase chain reaction-restriction fragment length polymorphism
Chinese Pharmacopoeia: Pharmacopoeia of the People’s Republic of China
HKCMMS: Hong Kong Chinese Materia Medica Standards
ITS: Internal transcribed spacer
rDNA: Ribosomal DNA
NIFDC: National Institutes for Food and Drug Control
ASVs: Amplicon sequence variants
