# Supplementary material for: Determination of ITS1 haplotypes of Fritillariae Cirrhosae Bulbus by amplicon sequencing
Source: Chin Med. 2024 Feb 28;19:33. doi: 10.1186/s13020-024-00911-3 (PMC10900738; doi:10.1186/s13020-024-00911-3)
Supplement: Supplementary file 1 — Additional file 1: Fig. S1. a) Maximum likelihood tree of ITS1 sequences from Fritillaria major ASVs and the T5177 reference sequence (highlighted in green) as well as 7 Lilium species (highlighted in orange); b) Loci of SmaI recognition site CCCGGG and its mutated forms of CTCGGG, CCACGGG and others, indicated by green, yellow, blue and grey, respectively; c) heatmap showing presence or absence of major ASVs in Fritillariae Cirrhosae Bulbus (FCB) and non-FCB species. F. hupehensis was found to be carried major ASVs with SmaI recognition site CCCGGG, which was due to the misidentification of T4940 as F. hupehensis or T4940 was mixed into a batch of F. hupehensis sample; d) heatmap showing the relative abundance of major ASVs in each FCB and non-FCB species. [file 13020_2024_911_MOESM1_ESM.docx]

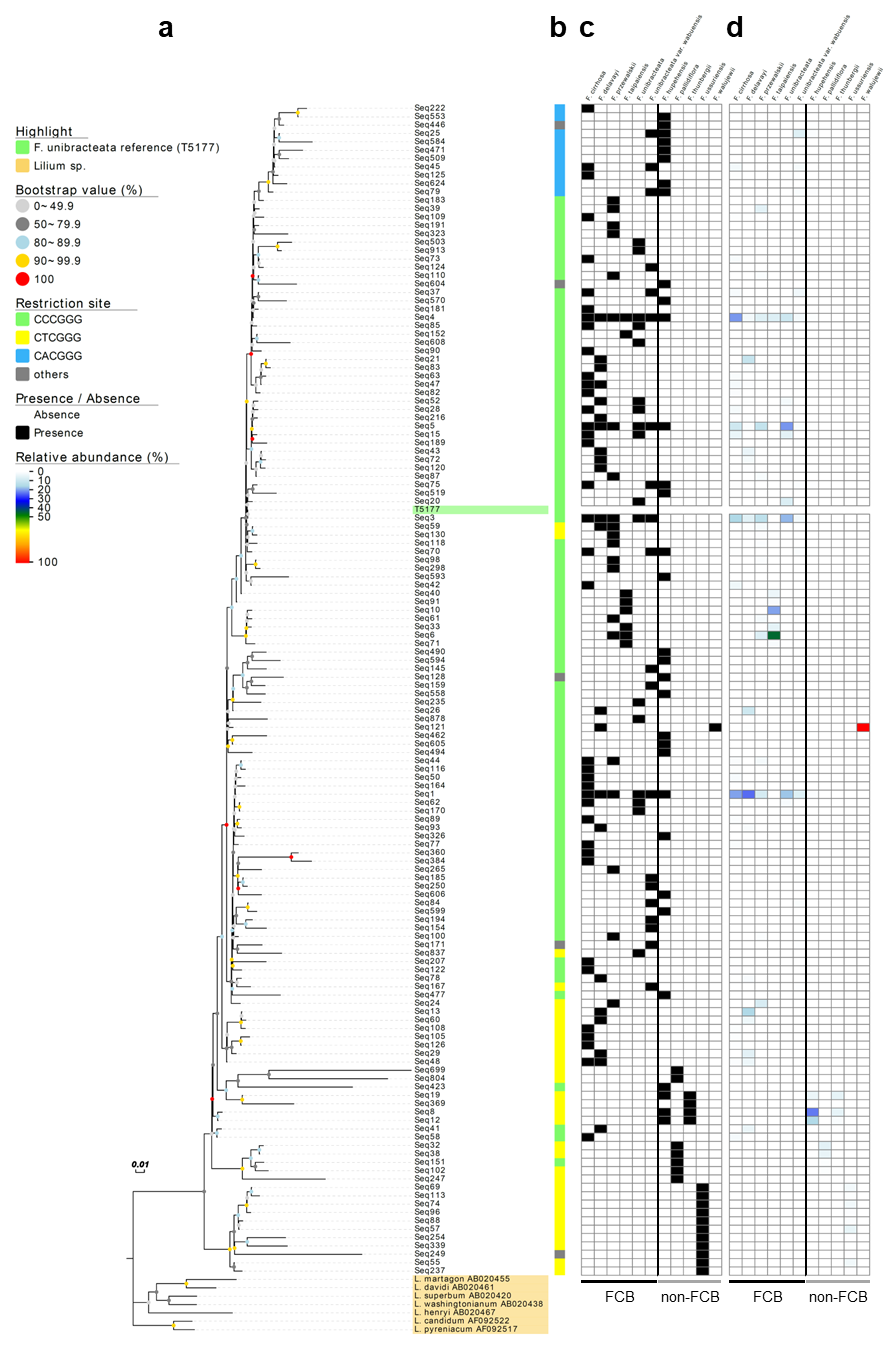


Supplementary Fig. 1

Fig. S1. a) Maximum likelihood tree of ITS1 sequences from *Fritillaria* major ASVs and the T5177 reference sequence (highlighted in green) as well as 7 *Lilium* species (highlighted in orange); b) Loci of *Sma*I recognition site CCCGGG and its mutated forms of CTCGGG, CCACGGG and others, indicated by green, yellow, blue and grey, respectively; c) heatmap showing presence or absence of major ASVs in Fritillariae Cirrhosae Bulbus (FCB) and non-FCB species. *F. hupehensis* was found to be carried major ASVs with *Sma*I recognition site CCCGGG, which was due to the misidentification of T4940 as *F. hupehensis* or T4940 was mixed into a batch of *F. hupehensis* sample; d) heatmap showing the relative abundance of major ASVs in each FCB and non-FCB species.
