# Supplementary material for: Determination of ITS1 haplotypes of Fritillariae Cirrhosae Bulbus by amplicon sequencing
Source: Chin Med. 2024 Feb 28;19:33. doi: 10.1186/s13020-024-00911-3 (PMC10900738; doi:10.1186/s13020-024-00911-3)
Supplement: Supplementary file 2 — Additional file 2: Fig. S2. Sanger sequencing electropherograms of the SmaI restriction site in the ITS1 region of Fritillaria Cirrhosae Bulbus (FCB) species. Minor variants in SmaI restriction sites (CCCGGG) of selected FCB samples, including RD188 of F. cirrhosa (a), T4975 of F. unibracteata var. wabuensis (b), T5233 of F. przewalskii (c), T5234 of F. delavayi (d) and T5236 of F. delavayi (e), were observed in the corresponding electropherograms. The minor peaks shown are in line with the high throughput sequencing results reported in Table 2. [file 13020_2024_911_MOESM2_ESM.docx]

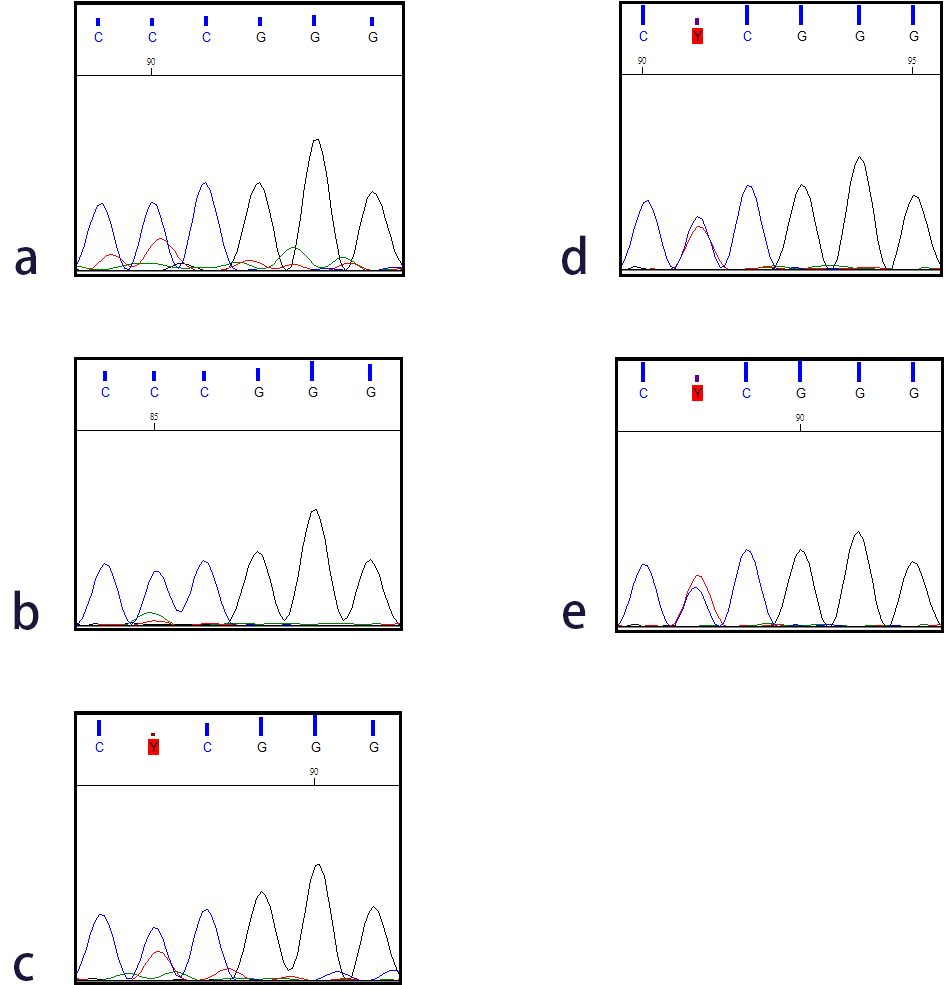


Fig. S2. Sanger sequencing electropherograms of the *Sma*I restriction site in the ITS1 region of Fritillaria Cirrhosae Bulbus (FCB) species. Minor variants in *Sma*I restriction sites (CCCGGG) of selected FCB samples, including RD188 of *F. cirrhosa* (a), T4975 of *F. unibracteata* var. *wabuensis* (b), T5233 of *F. przewalskii* (c), T5234 of *F. delavayi* (d) and T5236 of *F. delavayi* (e), were observed in the corresponding electropherograms. The minor peaks shown are in line with the high throughput sequencing results reported in Table 2.
